# Homomorphisms of Lattice-Valued Intuitionistic Fuzzy Subgroup Type-3

**DOI:** 10.1155/2022/6847138

**Published:** 2022-04-28

**Authors:** Sajida Kousar, Tahzeeb Saleem, Nasreen Kausar, Dragan Pamucar, Gezahagne Mulat Addis

**Affiliations:** ^1^Department of Mathematics and Statistics, International Islamic University Islamabad, Islamabad, Pakistan; ^2^Department of Mathematics, Faculty of Arts and Sciences, Yildiz Technical University, Esenler 34210, Istanbul, Turkey; ^3^Department of Logistics, University of Defence in Belgrade, Belgrade, Serbia; ^4^Department of Mathematics, University of Gondar, P.O. Box: 196, Gondar, Ethiopia

## Abstract

The lattice-valued intuitionistic fuzzy set was introduced by Gerstenkorn and Tepavcevi as a generalization of both the fuzzy set and the *L*-fuzzy set by incorporating membership functions, nonmembership functions from a nonempty set *X* to any lattice *L*, and lattice homomorphism from *L* to the interval [0,1]. In this article, lattice-valued intuitionistic fuzzy subgroup type-3 (LIFSG-3) is introduced. Lattice-valued intuitionistic fuzzy type-3 normal subgroups, cosets, and quotient groups are defined, and their group theocratic properties are compared with the concepts in classical group theory. LIFSG-3 homomorphism is established and examined in relation to group homomorphism. The research findings are supported by provided examples in each section.

## 1. Introduction

In the sixteenth century, Gerolamo Cardano laid the notion of probability theory to analyze games of chance. In the early nineteenth century, Pierre Laplace complied with the classical interpretation of probability that was assumed to be the best tool to deal with uncertainties in the experimental data. But there are several situations where uncertainty occurs as a vagueness more than a statistical variation. In 1965, Zadeh [1] presented a new concept of the fuzzy subset to carter the situation where probability fails to answer. The fuzzy subset of a nonempty set *U* as described by Zadeh is based on the formulation of a function *μ* from *U* to the closed interval [0,1]. The function is called a membership function, whereas the images of elements of *U* under this function are called membership grades. For instance, let *U* be a collection of finite groups, *p*(*x*) be the total number of subgroups in *x* ∈ *U*. If *q*(*x*) is the total number of normal subgroups computed by a student in *x*, then *μ*(*x*)=*q*(*x*)/*p*(*x*) defines a fuzzy membership grade to the normal subgroups in *x*. But there is a chance that if the group order is large and the student is unable to compute all the normal subgroups, then *μ*(*x*) will be greater than the one reported by the student. This leads us toward the concept of nonmembership grades first introduced by Atanassov [2], and the fuzzy set that incorporates membership and non-membership grades is termed an intuitionistic fuzzy set (IFS). Atanassov [3] presented basic models, properties, arithmetic operations, algebraic operators, and relations over the intuitionistic fuzzy set.

Over the years, several other generalizations of fuzzy sets have been introduced depending upon various parameters of uncertainty, vagueness, and imprecision by employing membership, nonmembership, hesitancy, and indeterminacy grades. In all these generalizations, the grades are real numbers ranging between 0 and 1. The interval [0,1] inherits the natural partial order from the set of real numbers and constitutes a lattice. Partial ordering and fuzzy uncertainties are key features of real-life problems with infinite solutions or no solution at all. So it is quite obvious to think about the replacement of [0,1] by any suitable lattice. Goguen [4] introduced the concept of *L*-fuzzy subsets of *S* where the interval [0,1] is replaced by a partially ordered set *L*. Atanassov [5] presented the concept of the lattice-valued intuitionistic fuzzy set (LIFS-1) by using a complete lattice *L*, an involutive order reversing unary operation *N* : *L*⟶*L* two functions *μ*, *ν* : *S*⟶*L*. Due to the compulsion of the operator *N*, the definition of LIFS-1 is not applicable to a larger collection of lattices. Gerstenkorn and Tepa $\overset{ˇ}{v}$ cev $\overset{´}{i}$ [6] refined the concept introduced by Atanassov. They replaced lattice with complete lattice and unary operator *N* with a linearization function *ℓ* : *L*⟶[0,1]; and termed their finding lattice-valued intuitionistic fuzzy set of a second type (LIFS-2). Different properties, such as the decomposition theorem and synthesis, were established for these fuzzy sets. However, the choice of a linearization map makes LIFS-2 less capable of dealing with basic set operations. For instance, the union of two LIFS-2s need not be a LIFS-2. Thus, the map was replaced with lattice homomorphism *α* : *L*⟶[0,1], and the refinement is called a lattice-valued intuitionistic fuzzy set type-3 and abbreviated as LIFS-3.

The term group was first used by Évariste Galois in the 1830s for the set of roots of polynomial equations. However, the modern-day definition of the group was established in 1870. Since then, significant research has been carried out in this area, and now the group is one of the most important algebraic structures providing a basic structure for several mathematical branches including analysis, game theory, coding theory, and algebraic geometry. Groups have strong applications in different scientific fields, especially symmetric groups, which play a vital role in theocratical physics and quantum mechanics. In genetics, the four-codon basis constitutes a group isomorphic to the Klein four-group. Gene mutation can be identified by establishing group homomorphism on copies of the sixty-four codon system. The coset diagram depicting group action has a close link with the crystal structure in chemistry. After Zadeh's invention, many researchers attempted to use and replace the ordinary set with the fuzzy set in various theocratical and experimental areas.

In 1970, Rosenfeld [7] attempted to combine fuzzy concepts in group theory and termed the findings as a fuzzy subgroup. Rosenfeld investigated fundamental group theocratic properties for the newly established algebra. In later years, algebraists examined the structural properties of fuzzy subgroups. Anthony [8, 9] modified the definition of Rosenfeld by strengthening the condition for images of elements and their inverses. In fuzzy groups, it is observed that level sets and proved that a fuzzy subset of a group *G* is a fuzzy subgroup if and only if all the level sets are subgroups of *G* [10, 11]. In 1982, Liu [12] suggested fuzzy invariant subgroups and fuzzy ideals. Ajmal and Prajapati [13] and Mukherjee et al. [14, 15] connected fuzzy normal subgroups and fuzzy cosets and group-theoretic analogs. Kumar et al. [16] resolved fuzzy normal subgroups and fuzzy quotients. Moreover, Tarnauceanu [17] presented the concept of fuzzy normal subgroups for the class of finite groups. Choudhary et al. [18] and Addis [19] investigated structure-preserving maps and fundamental isomorphism theorems. Malik et al. [20] and Mishref [21] introduced the fuzzy normal series to generalize the concept of nilpotancy and solubility of groups to fuzzy subgroups. Zhan and Zhisong [22] defined the intuitionistic fuzzy subgroup as a generalization of Rosenfeld's fuzzy subgroup. By starting with a given classical group, they define a intuitionistic fuzzy subgroup using the classical binary operation defined over the given classical group. Li and Gui [23] extended Zhan and Zhisong's work on intuitionistic fuzzy groups. Tarsuslu et al. [24] generalized the action of a group on a set to intuitionistic fuzzy action. Bal et al. [25] investigated the kernel subgroup of intuitionistic fuzzy subgroups.

The employment of lattice order turns the *L*-fuzzy set into an important generalization of the fuzzy set widely applicable in decision language [4], system analysis [26], and coding theory [27]. Group theory is essential not only for mathematical advancements, but also for other scientific fields such as physics [28–30] and chemistry [31, 32]. Since 1970, several mathematicians have been extensively investigated group structure in fuzzy and generalized fuzzy environments. The importance of LIFS-3 and group on their own motivates to combine these two concepts. The main objective of this article is to introduce the notion of lattice-valued intuitionistic fuzzy subgroup type-3 and analyze its algebraic properties.

## 2. Preliminaries

This section is introductory in nature and contains all the essential definitions and fundamental properties that are necessary to understand the newly established structure of LIFSG-3.

### 2.1. L-Fuzzy Subset

To understand a *L*-fuzzy subset [4], first we will define the order relation and lattice. For a nonempty set *P*, a reflexive, antisymmetric, and transitive relation is termed as a partial order on *P*, commonly denoted by the notation “less *L* than or equal,” that is, ≤. The set *P* is termed as a partially ordered set. If *P* is a partially ordered set, then the partial order gives an instinct to the concept of the greatest and smallest element in the set. If *a*, *b* ∈ *P* are related in such a way that *a* ≤ *b*, then we can pronounce it as *b* is greater than *a* or another way round *a* is smaller than *b*.

Now if ∅≠*Q* is any subset of *P*, then a member ϱ of *P* is called an upper (lower) bound of *Q*if ϱ is greater (smaller) than all the elements in *Q*. Perhaps *Q* has more than one upper (lower) bounds, the smallest (greatest) of these upper (lower) bounds is termed to be supremum or meet (infimum or join) of *Q*, denoted by ∨(∧). A set *L* together with partial order in which join (∧) and meet (∨) exist for every pair of elements is called a lattice, denoted by (*L*, ≤, ∨, ∧). A lattice *L* is said to be complete if ∨ and ∧ exits for every nonempty subset of *L*. A lattice *L* is titled to be distributive if ∨ and ∧ are distributive over each other.

**Definition 1 .**

Let *𝒮* be a nonempty set and *L*=(*L*, ≤, ∨, ∧) be a complete distributive lattice which has least (bottom) and greatest (top) elements say *B* and *T*, respectively. Then, an *L*-fuzzy subset $\zeta_{\tilde{\overset{´}{𝒮}}}$ of *𝒮* is narrated as follows:

(1) $$\begin{array}{c} \zeta_{\tilde{\overset{´}{S}}}:S⟶L. \end{array}$$

**Definition 2 .**

If *𝒮* is a group and the *L*-fuzzy subset satisfy the following conditions for all *s*_1_, *s*_2_ ∈ *𝒮*,

(2) $$\begin{array}{c} \zeta_{\tilde{\overset{´}{S}}}\left(s_{1}s_{2}\right)\geq \wedge \left\{\zeta_{\tilde{\overset{´}{S}}}\left(s_{1}\right),\zeta_{\tilde{\overset{´}{S}}}\left(s_{2}\right)\right\},\\ \zeta_{\tilde{\overset{´}{\overset{´}{S}}}}\left(s^{-1}\right)=\zeta_{\tilde{\overset{´}{\overset{´}{S}}}}\left(s\right)\;\forall s\in G. \end{array}$$

Then, it is termed as a *L*- fuzzy subgroup [33] of *𝒮*.

### 2.2. L-Intutionistic Fuzzy Subset

Gerstenkorn and Tepa $\overset{ˇ}{v}$ cev $\overset{´}{i}$ [6] refined the concept introduced by Atanassov. They replaced lattice by complete lattice; unary operator *N* by lattice homomorphism *℘* : *L*⟶[0,1] and refinement is abbreviated as (LIFS-3).

**Definition 3 .**

A LIFS-3 is the set (*𝒮*, *L*, *ζ*_*ℑ*_*L*__, *ξ*_*ℑ*_*L*__, *℘*), with *𝒮* a nonempty set, *L* is a complete lattice with top and bottom elements *T* and *B*, respectively, *ζ*_*ℑ*_*L*__ : *X*⟶*L* and *ξ*_*ℑ*_*L*__ : *𝒮*⟶*L* are membership and nonmemberships functions. The map *℘* : *L*⟶[0,1] is a lattice homomorphism with *℘*(*T*)=1, *℘*(*B*)=0(that is, for all *ℓ*_1_,  *ℓ*_2_ ∈ *L*,

(3) $$\begin{array}{c} \left.\begin{array}{c} ℘\left(\ell_{1}\wedge \ell_{2}\right)=℘\left(\ell_{1}\right)\wedge ℘\left(\ell_{2}\right)\\ ℘\left(\ell_{1}\vee \ell_{2}\right)=℘\left(\ell_{1}\right)\vee ℘\left(\ell_{2}\right) \end{array}\right). \end{array}$$

Such that for every *s* ∈ *S*, *℘*(*ζ*_*ℑ*_*L*__(*s*))+*℘*(*ξ*_*ℑ*_*L*__(*s*)) ≤ 1.

**Example 1 .**

Consider *S*={*a*, *b*, *c*, *d*, *e*, *f*} and the lattice *L*={*B*, *r*, *s*, *t*, *τ*} w.r.t partial order

(4) $$\begin{array}{c} B\leq B,\\ B\leq r,\\ B\leq s,\\ B\leq t,\\ B\leq \tau \\ \tau \leq \tau ,\\ B\leq \tau ,\\ r\leq \tau ,\\ s\leq \tau ,\\ t\leq \tau ,\\ r\leq s. \end{array}$$

Define *℘* : *L*⟶[0,1], *ζ*_*ℑ*_*L*__ : *S*⟶*L* and *ξ*_*ℑ*_*L*__ : *𝒮*⟶*L*

(5) $$\begin{array}{c} ℘\left(\tau \right)=1,\\ ℘\left(B\right)=0,\\ ℘\left(r\right)=0.2,\\ ℘\left(s\right)=0.5,\\ ℘\left(t\right)=0.3,\\ \zeta_{{ℑ}_{L}}\left(a\right)=\tau ,\\ \zeta_{{ℑ}_{L}}\left(b\right)=\zeta_{{ℑ}_{L}}\left(c\right)=s,\\ \zeta_{{ℑ}_{L}}\left(d\right)=\zeta_{{ℑ}_{L}}\left(e\right)=\zeta_{{ℑ}_{L}}\left(f\right)=r,\\ \zeta_{{ℑ}_{L}}\left(a\right)=\tau ,\\ \zeta_{{ℑ}_{L}}\left(b\right)=\zeta_{{ℑ}_{L}}\left(c\right)=s,\\ \zeta_{{ℑ}_{L}}\left(d\right)=\zeta_{{ℑ}_{L}}\left(e\right)=\zeta_{{ℑ}_{L}}\left(f\right)=r. \end{array}$$

Then, for every *s* ∈ *S*, *℘*(*ζ*_*ℑ*_*L*__(*s*))+*℘*(*ξ*_*ℑ*_*L*__(*s*)) ≤ 1 imply that (*𝒮*, *L*, *ζ*_*ℑ*_*L*__, *ξ*_*ℑ*_*L*__, *℘*) is a LIFS-3 of *𝒮*.

## 3. Lattice-Valued Intuitionistic Fuzzy Subgroup Type-3

We will define lattice-valued intuitionistic fuzzy subgroups (LIFSG) by using a group *G*, membership *ζ*_*I*_*L*__  :  *G* ⟶ *L*, nonmembership *ξ*_*I*_*L*__  :  *G* ⟶ *L*, and a lattice homomorphism *℘*  :  *L* ⟶ *L*. The combination of LIFS − 3 and group will provide us a new refined algebraic structure which we can use effectively in real world problems.

**Definition 4 .**

For a group *G*, lattice *L* with top and bottom elements *T* and *B*, respectively, and lattice homomorphism *℘* : *L*⟶[0,1]. A LIFS-3 of *G*, that is, *I*_*L*_ = (*G*, *L*, *ζ*_*I*_*L*__, *ξ*_*I*_*L*__, *℘*) formulates a LIFSG-3 of *G* provided that for every *x*, *y* ∈ *G*,

1. *ζ*_*I*_*L*__(*xy*) ≥ *ζ*_*I*_*L*__(*x*)∧*ζ*_*I*_*L*__(*y*) and *ξ*_*I*_*L*__(*xy*)  ≤  *ξ*_*I*_*L*__(*x*)∨*ξ*_*I*_*L*__(*y*)
2. *ζ*_*I*_*L*__(*x*)=*ζ*_*I*_*L*__(*x*^−1^) and *ξ*_*I*_*L*__(*x*)=*ξ*_*I*_*L*__(*x*^−1^)

**Proposition 1 .**

Let *L* be a lattice (with top and bottom elements *T* and *B*, respectively) and *G* be a group. Let *I*_*L*_ = (*G*, *L*, *ζ*_*I*_*L*__, *ξ*_*I*_*L*__, *℘*) be a LIFSG-3 of *G*. For *t*_1_  ∈  Im*ζ*_*I*_*L*__ and *t*_2_  ∈  Im*ξ*_*I*_*L*__, the (*t*_1_, *t*_2_)-cut set (or level set) is a subgroup of *G*, called (*t*_1_, *t*_2_)-cut subgroup (or level subgroup).

**Proof**

Recall the definition of cut sets in IFS where these sets are defined as follows:

(6) $$\begin{array}{c} I_{L}^{\left(t_{1},t_{2}\right)}=\left\{x\in G:\zeta_{I_{L}}\left(x\right)\geq t_{1}\text{ and }\xi_{I_{L}}\left(x\right)\leq t_{2}\right\}. \end{array}$$

Let *e* be the identity element in *G*. Then, for any *u* ∈ *G* *e* ∈ *uu*^−1^

(7) $$\begin{array}{c} \Rightarrow \zeta_{I_{L}}\left(e\right)=\zeta_{I_{L}}\left(uu^{-1}\right)\geq \zeta_{I_{L}}\left(u\right)\wedge \zeta_{I_{L}}\left(u^{-1}\right). \end{array}$$

By the property of LIFSG, we have

(8) $$\begin{array}{c} \zeta_{I_{L}}\left(e\right)=\zeta_{I_{L}}\left(uu^{-1}\right)\geq \zeta_{I_{L}}\left(u\right)\wedge \zeta_{I_{L}}\left(u\right),\\ \Rightarrow \zeta_{I_{L}}\left(e\right)\geq \zeta_{I_{L}}\left(u\right)\forall u\in G. \end{array}$$

⇒ *ζ*_*I*_*L*__(*e*) is an upper bound for Im*ζ*_*I*_*L*__.

Similarly, *ξ*_*I*_*L*__(*e*) ≤ *ξ*_*I*_*L*__(*u*)∀ *u* ∈ *G*, this implies that *ξ*_*I*_*L*__(*e*) is an lower bound for Im *ξ*_*I*_*L*__. We get that *e* ∈ *I*_*L*_^(*t*_1_, *t*_2_)^. Let *u*, *v* ∈ *I*_*L*_^(*t*_1_, *t*_2_)^. Then,

(9) $$\begin{array}{c} \zeta_{I_{L}}\left(u\right),\;\zeta_{I_{L}}\left(v\right)\geq t_{1},\\ \xi_{I_{L}}\left(u\right),\;\xi_{I_{L}}\left(v\right)\leq t_{2},\\ \zeta_{I_{L}}\left(uv^{-1}\right)\geq \zeta_{I_{L}}\left(u\right)\wedge \xi_{I_{L}}\left(v^{-1}\right),\\ \Rightarrow \zeta_{I_{L}}\left(uv^{-1}\right)\geq t_{1}. \end{array}$$

Similarly, *ξ*_*I*_*L*__(*uv*^−1^) ≤ *t*_2_, this implies that *uv*^−1^ ∈ *I*_*L*_^(*t*_1_, *t*_2_)^. Hence, *I*_*L*_^(*t*_1_, *t*_2_)^ is a subgroup of *G*.

**Proposition 2 .**

For a group *G* and lattice *L*, let *I*_*L*_=(*G*, *L*, *ζ*_*I*_*L*__, *ξ*_*I*_*L*__, *℘*) be a LIFS-3 such that for each *t*_1_, *t*_2_  ∈  *L*, the (*t*_1_, *t*_2_)-cut set *I*_*L*_^(*t*_1_, *t*_2_)^ ≤ *G*. Then, *I* is a LIFSG-3 of *G*.

**Proof**

Suppose for all *t*_1_ ∈ Im *ζ*_*I*_*L*__,  *t*_2_ ∈ Im *ξ*_*I*_*L*__, *I*_*L*_^(*t*_1_, *t*_2_)^ ≤ *G*. Let *u*, *v*  ∈  *G*. Then, there are two possibilities

- Case 1: Let *u*, *v* ∈ *I*_*L*_^(*t*_1_, *t*_2_)^. Then, *uv* ∈ *I*_*L*_^(*t*_1_, *t*_2_)^ and

(10) $$\begin{array}{c} \zeta_{I_{L}}\left(u\right)\geq t_{1},\;\zeta_{I_{L}}\left(v\right)\geq t_{1},\\ \xi_{I_{L}}\left(u\right)\leq t_{2},\;\xi_{I_{L}}\left(v\right)\leq t_{2}\\ \Rightarrow \zeta_{I_{L}}\left(uv\right)\geq t_{1},\xi_{I_{L}}\left(uv\right)\leq t_{2}. \end{array}$$

  Suppose *ζ*_*I*_*L*__(*u*)=*ℓ*_1_ and *ζ*_*I*_*L*__(*v*)=*ℓ*_2_ and *ℓ*_3_=*ℓ*_1_∧*ℓ*_2_. Then *ℓ*_3_ ≤ *ℓ*_1_ and *ℓ*_2_ and *uv* ∈ *I*_*L*_^(*ℓ*_3_, *t*_2_)^. We get that

(11) $$\begin{array}{c} \zeta_{I_{L}}\left(uv\right)\geq \ell_{3}=\zeta_{I_{L}}\left(u\right)\wedge \zeta_{I_{L}}\left(v\right). \end{array}$$

  Similarly, suppose $\xi_{I_{L}}\left(u\right)=\ell_{4}\sqrt{2}$ and *ξ*_*I*_*L*__(*v*)=*ℓ*_5_ and *ℓ*_6_=*ℓ*_4_∨*ℓ*_5_. Then, *ℓ*_6_ ≥ *ℓ*_4_ and *ℓ*_5_ and *uv* ∈ *I*_*L*_^(*t*_1_, *ℓ*_6_)^. We get that

(12) $$\begin{array}{c} \xi_{I_{L}}\left(uv\right)\leq \ell_{6}=\xi_{I_{L}}\left(u\right)\vee \xi_{I_{L}}\left(v\right). \end{array}$$

  If *u* ∈ *I*_*L*_^(*t*_1_, *t*_2_)^ ≤ *G*, then by definition *u*^−1^ ∈ *I*_*L*_^(*t*_1_, *t*_2_)^. Now using the same argument as above it is easy to show that

(13) $$\begin{array}{c} \zeta_{I_{L}}\left(u^{-1}\right)=\zeta_{I_{L}}\left(u\right),\\ \xi_{I_{L}}\left(u^{-1}\right)=\xi_{I_{L}}\left(u\right). \end{array}$$

(ii) Case 2: if *u* ∈ *I*_*L*_^(*t*_1_, *t*_2_)^,  *v* ∈ *I*_*L*_^(*t*_3_, *t*_4_)^, then three cases arise:
  1. *t*_1_  ≤  *t*_3_,  *t*_4_  ≤  *t*_2_, then *I*_*L*_^(*t*_3_, *t*_4_)^ ≤ *I*_*L*_^(*t*_1_, *t*_2_)^, this implies that *u*, *v* ∈ *I*_*L*_^(*t*_1_, *t*_2_)^
  2. *t*_1_  ≤  *t*_3_,  *t*_2_  ≤  *t*_4_. As *L* is a lattice so *t*_2_ ∨ *t*_4_ exist. Suppose *t*_5_ = *t*_2_ ∨ *t*_4_ then *t*_2_, *t*_4_  ≤  *t*_5_

(14) $$\begin{array}{c} I_{L}^{\left(t_{1},t_{5}\right)}=\left\{g\in G:\zeta_{I_{L}}\left(g\right)\geq t_{1},\xi_{I_{L}}\left(g\right)\leq t_{5}\right\}. \end{array}$$

Now, *ζ*_*I*_*L*__(*v*) ≥ *t*_3_ ≥ *t*_1_,  *ξ*_*I*_*L*__(*u*) ≤ *t*_2_ ≤ *t*_5_,  *ξ*_*I*_*L*__(*v*) ≤ *t*_4_ ≤ *t*_5_
  (15) $$\begin{array}{c} \Rightarrow \;u,v\;\in I_{L}^{\left(t_{1},t_{5}\right)}. \end{array}$$
(3) *t*_1_  ≥  *t*_3_,  *t*_4_  ≤  *t*_2_. As *L* is a lattice so *t*_1_ ∧ *t*_3_ exist. Suppose *t*_6_ = *t*_1_ ∧ *t*_3_ then *t*_1_, *t*_3_  ≥  *t*_6_,
  (16) $$\begin{array}{c} I_{L}^{\left(t_{6},t_{4}\right)}=\left\{g\in G:\zeta_{I}\left(g\right)\geq t_{6},\xi_{I}\left(g\right)\leq t_{4}\right\}. \end{array}$$

Now *ζ*_*I*_*L*__(*v*) ≥ *t*_3_ ≥ *t*_6_,  *ζ*_*I*_*L*__(*u*) ≥ *t*_1_ ≥ *t*_6_,  *ξ*_*I*_*L*__(*v*) ≤ *t*_4_ ≤ *t*_2_

(17) $$\begin{array}{c} \Rightarrow \;u,v\in I_{L}^{\left(t_{6},t_{2}\right)}. \end{array}$$

On the basis of previous discussion we conclude that the elements of *G* sustain the axioms of LIFSG-3. Hence, *I*_*L*_ is a LIFSG-3 of *G*.

**Remark 1 .**

For every group *G* and lattice *L* with *T* and *B* as the top and bottom element, the LIFS-3 *I*_*L*_=(*G*, *L*, *ζ*_*I*_*L*__, *ξ*_*I*_*L*__, *℘*) is a LIFSG-3 of *G* if and only if

(18) $$\begin{array}{c} I_{L}^{\left(t_{1},t_{2}\right)}\leq G\;\forall t_{1},t_{2}\in L. \end{array}$$

**Proposition 3 .**

For a group *G* and lattice *L* with *T* and *B* as the top and bottom element, let *H*  ≤  *G*. For *t* ∈ *L*, define *ζ*_*H*_*t*__ and *ξ*_*H*_*t*__ from *G* to *L* as

(19) $$\begin{array}{c} \zeta_{H_{t}}\left(g\right)=\left\{\begin{array}{cc} t, & \text{if }g\in H,\\ B, & \text{if }g\in \frac{G}{H}, \end{array}\right.\\ \xi_{H_{t}}\left(g\right)=\left\{\begin{array}{cc} t, & \text{if }g\in H,\\ T, & \text{if }g\in \frac{G}{H}. \end{array}\right. \end{array}$$

Let *℘* : *L*⟶[0,1] be a lattice homomorphism. Then, *℘*(*T*)=1 and *℘*(*B*)=0. Suppose *℘*(*t*)=0.2, then ${\dot{I}}_{L}=\left(G,L,\zeta_{H_{t}},\xi_{H_{t}},℘\right)$ is a LIFSG-3 of *G*.

**Proof**

Let *℘* : *L*⟶[0,1] be a lattice homomorphism defined as *℘*(*T*)=1 and *℘*(*B*)=0 and *℘*(*t*)=0.2. For *g*  ∈  *G* we have two cases:

1. Case 1: Let *g*  ∈  *H*, *ζ*_*H*_*t*__(*g*)=*t*,  *ξ*_*H*_*t*__(*g*)=*t*

(20) $$\begin{array}{c} \Rightarrow ℘\left(\zeta_{H_{t}}\left(g\right)\right)+℘\left(\xi_{H_{t}}\left(g\right)\right)=℘\left(t\right)+℘\left(t\right)=0.4<1. \end{array}$$

(2) Case 2: Let *g* ∈ *G*/*H*, *ζ*_*H*_*t*__(*g*)=*B*,  *ξ*_*H*_*t*__(*g*)=*T*

(21) $$\begin{array}{c} \Rightarrow ℘\left(\zeta_{H_{t}}\left(g\right)\right)+℘\left(\xi_{H_{t}}\left(g\right)\right)=℘\left(B\right)+℘\left(T\right)=1. \end{array}$$

Now, for *g*_1_, *g*_2_  ∈  *G*, we have three cases:

1. *g*_1_, *g*_2_  ∈  *H*, this implies that *g*_1_*g*_2_  ∈  *H*

(22) $$\begin{array}{c} \zeta_{H_{t}}\left(g_{1}\right)=t,\\ \zeta_{H_{t}}\left(g_{2}\right)=t,\\ \zeta_{H_{t}}\left(g_{1}g_{2}\right)=t,\\ \xi_{H_{t}}\left(g_{1}\right)=t,\\ \xi_{H_{t}}\left(g_{2}\right)=t,\\ \xi_{H_{t}}\left(g_{1}g_{2}\right)=t. \end{array}$$

(2) *g*_1_ ∈ *H*, *g*_2_ ∈ *G*/*H*, this implies that *g*_1_*g*_2_  ∉  *H*

(23) $$\begin{array}{c} \Rightarrow g_{1}g_{2}\in G/H. \end{array}$$

  Thus, *ζ*_*H*_*t*__(*g*_1_)=*t*, *ζ*_*H*_*t*__(*g*_2_)=*B*, *ζ*_*H*_*t*__(*g*_1_*g*_2_)=*B*

(24) $$\begin{array}{c} \Rightarrow \;\zeta_{H_{t}}\left(g_{1}\right)\wedge \zeta_{H_{t}}\left(g_{2}\right)=t\wedge B=B,\\ \Rightarrow \zeta_{H_{t}}\left(g_{1}g_{2}\right)=B=\zeta_{H_{t}}\left(g_{1}\right)\wedge \zeta_{H_{t}}\left(g_{2}\right). \end{array}$$

Similarly, *ξ*_*H*_*t*__(*g*_1_*g*_2_)=*T*=*ξ*_*H*_*t*__(*g*_1_)∨*ξ*_*H*_*t*__(*g*_2_).
(3) *g*_1_, *g*_2_ ∈ *G*/*H*, then two cases arises:
  - *g*_1_*g*_2_ ∈ *G*/*H*

(25) $$\begin{array}{c} \zeta_{H_{t}}\left(g_{1}\right)=B,\zeta_{H_{t}}\left(g_{2}\right),\\ =B,\zeta_{H_{t}}\left(g_{1}g_{2}\right),\\ =B\xi_{H_{t}}\left(g_{1}\right),\\ =T,\\ \xi_{H_{t}}\left(g_{2}\right)=T,\\ \xi_{H_{t}}\left(g_{1}g_{2}\right)=T. \end{array}$$

(b) *g*_1_*g*_2_ ∈ *H*
  (26) $$\begin{array}{c} \zeta_{H_{t}}\left(g_{1}\right)=B,\zeta_{H_{t}}\left(g_{2}\right)=B,\zeta_{H_{t}}\left(g_{1}g_{2}\right)=t\geq B\xi_{H_{t}}\left(g_{1}\right)\\ =T,\xi_{H_{t}}\left(g_{2}\right)=T,\xi_{H_{t}}\left(g_{1}g_{2}\right)=t\leq T. \end{array}$$

From previous discussion we get that

(27) $$\begin{array}{c} \zeta_{H_{t}}\left(g_{1}g_{2}\right)\geq \zeta_{H_{t}}\left(g_{1}\right)\wedge \zeta_{H_{t}}\left(g_{2}\right),\\ \xi_{H_{t}}\left(g_{1}g_{2}\right)\leq \xi_{H_{t}}\left(g_{1}\right)\vee \xi_{H_{t}}\left(g_{2}\right),\\ \zeta_{H_{t}}\left(g\right)=\zeta_{H_{t}}\left(g^{-1}\right),\;\\ \xi_{H_{t}}\left(g\right)=\xi_{H_{t}}\left(g^{-1}\right). \end{array}$$

$\Rightarrow \;{\dot{I}}_{L}$ is a lattice-valued intuitionistic fuzzy subgroup type-3 of *G*.

Now, if *s*_1_ ∈ Im *ζ*_*H*_*t*__, *s*_2_ ∈ Im *ξ*_*H*_*t*__

(28) $$\begin{array}{c} \Rightarrow s_{1}\in \left\{B,t\right\},\;s_{2}\in \left\{t,T\right\}. \end{array}$$

Then, (*t*, *t*)− cut subgroup

(29) $$\begin{array}{c} {\dot{I}}_{L}^{\left(t,t\right)}=\left\{g\in G:\zeta_{H_{t}}\left(g\right)\geq t,\;\xi_{H_{t}}\left(g\right)\leq t\right\},\\ =H. \end{array}$$

**Remark 2 .**

Every subgroup *H* of *G* is a cut subgroup of some suitable LIFSG-3 of *G*.

## 4. Lattice-Valued Intuitionistic Fuzzy Normal Subgroups Type-3

As we defined the normality of the intuitionistic fuzzy subgroup in the previous chapter, similarly, in this chapter, we construct the lattice-valued intuitionistic fuzzy normal subgroup type-3 (LIFNSG-3). Then, using this definition, we proved some useful results in this section.

**Definition 5 .**

For a group *G*, lattice *L* with top element *T* and bottom element *B* and lattice homomorphism *℘* : *L*⟶[0,1]. Let *I*_*L*_=(*G*, *L*, *ζ*_*I*_*L*__, *ξ*_*I*_*L*__, *℘*) be a LIFSG-3. Then, *I*_*L*_ is called LIFNSG-3 *G* if

(30) $$\begin{array}{c} \zeta_{I_{L}}\left(xyx^{-1}\right)=\zeta_{I_{L}}\left(y\right),\\ \xi_{I_{L}}\left(xyx^{-1}\right)=\xi_{I_{L}}\left(y\right)\forall x,\;y\in G. \end{array}$$

**Proposition 4 .**

For a group *G*, lattice *L* with top element *T* and bottom element *B* and lattice homomorphism *℘* : *L*⟶[0,1]. If *I*_*L*_=(*G*, *L*, *ζ*_*I*_*L*__, *ξ*_*I*_*L*__, *℘*) is a LIFNSG-3 of *G*, then for *t*_1_ ∈ Im *ζ*_*I*_*L*__ and *t*_2_ ∈ Im *ξ*_*I*_*L*__

(31) $$\begin{array}{c} I_{L}^{\left(t_{1},t_{2}\right)}\underline{⊲}G. \end{array}$$

**Proof**

Suppose *I*_*L*_ is a LIFNSG-3 of *G*. Let *y*  ∈  *I*_*L*_^*t*_1_,*t*_2_^ and *x*  ∈  *G*. Then, *ζ*_*I*_*L*__(*y*) ≥ *t*_1_ and *ξ*_*I*_*L*__(*y*) ≤ *t*_2_ this implies that

*xyx* ^−1^ ∈ *I*_*L*_^(*t*_1_, *t*_2_)^,

(32) $$\begin{array}{c} \Rightarrow I_{L}^{\left(t_{1},t_{2}\right)}\underline{⊲}G. \end{array}$$

**Proposition 5 .**

For a group *G*, the set *I*_*L*_=(*G*, *L*, *ζ*_*I*_*L*__, *ξ*_*I*_*L*__, *℘*) is a LIFNSG-3 of *G* if and only if

(33) $$\begin{array}{c} \zeta_{I_{L}}\left(xy\right)=\zeta_{I_{L}}\left(yx\right),\\ \xi_{I_{L}}\left(xy\right)=\xi_{I_{L}}\left(yx\right)\forall x,\;y\in G. \end{array}$$

**Proof**

Suppose *I*_*L*_ is a LIFNSG-3 of *G*. Then, *ζ*_*I*_*L*__(*xyx*^−1^)=*ζ*_*I*_*L*__(*y*)∀ *x*, *y* ∈ *G*, implies that *ζ*_*I*_*L*__(*xy*)=*ζ*_*I*_*L*__(*yx*). Similarly we get that *ξ*_*I*_*L*__(*xy*)=*ξ*_*I*_*L*__(*yx*). Conversely, assume that *ζ*_*I*_*L*__(*xy*)=*ζ*_*I*_*L*__(*yx*) and *ξ*_*I*_*L*__(*xy*)=*ξ*_*I*_*L*__(*yx*)∀ *x*,  *y* ∈ *G*. Then, *ζ*_*I*_*L*__((*xy*)*x*^−1^)=*ζ*_*I*_*L*__(*x*^−1^(*xy*))=*ζ*_*I*_*L*__(*y*) and *ξ*_*I*_*L*__((*xy*)*x*^−1^)=*ξ*_*I*_*L*__(*x*^−1^(*xy*))=*ξ*_*I*_*L*__(*y*).

**Proposition 6 .**

Let *I*_*L*_=(*G*, *L*, *ζ*_*I*_*L*__, *ξ*_*I*_*L*__, *℘*) be a LIFSG-3 of *G*. Then *I*_*L*_ is LIFNSG-3 if and only if for all *x*, *y*  ∈  *G*,

(34) $$\begin{array}{c} \zeta_{I_{L}}\left(\left[x,y\right]\right)\geq \zeta_{I_{L}}\left(y\right),\\ \xi_{I_{L}}\left(\left[x,y\right]\right)\leq \xi_{I_{L}}\left(y\right). \end{array}$$

**Proof**

Suppose *I*_*L*_ is LIFNSG-3 of *G*. Then, *ζ*_*I*_*L*__([*x*, *y*])=*ζ*_*I*_*L*__(*xyx*^−1^*y*^−1^) implies that *ζ*_*I*_*L*__([*x*, *y*]) ≥ *ζ*_*I*_*L*__(*xyx*^−1^)∧*ζ*_*I*_*L*__(*y*^−1^). We get that *ζ*_*I*_*L*__([*x*, *y*]) ≥ *ζ*_*I*_*L*__(*y*). Similarly, *ξ*_*I*_*L*__([*x*, *y*]) ≤ *ξ*_*I*_*L*__(*y*). Conversely, assume that *ζ*_*I*_*L*__([*x*, *y*]) ≥ *ζ*_*I*_*L*__(*y*) and *ξ*_*I*_*L*__([*x*, *y*]) ≤ *ξ*_*I*_*L*__(*y*). Then *ζ*_*I*_*L*__(*xyx*^−1^)=*ζ*_*I*_*L*__(*xyx*^−1^*y*^−1^*y*). We get that *ζ*_*I*_*L*__(*xyx*^−1^) ≥ *ζ*_*I*_*L*__([*x*, *y*])∧*ζ*_*I*_*L*__(*y*).

(35) $$\begin{array}{c} \Rightarrow \zeta_{I_{L}}\left(xyx^{-1}\right)\geq \zeta_{I_{L}}\left(y\right). \end{array}$$

(36) $$\begin{array}{c} \xi_{I_{L}}\left(xyx^{-1}\right)\leq \xi_{I_{L}}\left(y\right). \end{array}$$

As *ζ*_*I*_*L*__(*y*)=*ζ*_*I*_*L*__(*x*^−1^*xyx*^−1^*x*) implies *ζ*_*I*_*L*__(*y*) ≥ *ζ*_*I*_*L*__(*x*)∧{*ζ*_*I*_*L*__(*xyx*^−1^)∧*ζ*_*I*_*L*__(*x*)}. If *ζ*_*I*_*L*__(*x*)∧*ζ*_*I*_*L*__(*xyx*^−1^)=*ζ*_*I*_*L*__(*x*), then *ζ*_*I*_*L*__(*y*) ≥ *ζ*_*I*_*L*__(*x*)∀ *x*, *y* ∈ *G*. ⇒ *ζ*_*I*_*L*__ is constant. In this case,

(37) $$\begin{array}{c} \zeta_{I_{L}}\left(y\right)=\zeta_{I_{L}}\left(xyx^{-1}\right). \end{array}$$

If *ζ*_*I*_*L*__(*x*)∧*ζ*_*I*_*L*__(*xyx*^−1^)=*ζ*_*I*_*L*__(*xyx*^−1^)

(38) $$\begin{array}{c} \Rightarrow \zeta_{I_{L}}\left(y\right)\geq \zeta_{I_{L}}\left(xyx^{-1}\right). \end{array}$$

If *ξ*_*I*_*L*__(*x*)∨*ξ*_*I*_*L*__(*xyx*^−1^)=*ξ*_*I*_*L*__(*x*), then this implies that *ξ*_*I*_*L*__(*y*) ≤ *ξ*_*I*_*L*__(*x*)∀ *x*, *y* ∈ *G*. Then, *ξ*_*I*_*L*__ is constant. In this case *ξ*_*I*_*L*__(*y*)=*ξ*_*I*_*L*__(*xyx*^−1^). If *ξ*_*I*_*L*__(*x*)∨*ξ*_*I*_*L*__(*xyx*^−1^)=*ξ*_*I*_*L*__(*xyx*^−1^)

(39) $$\begin{array}{c} \Rightarrow \;\xi_{I_{L}}\left(y\right)\leq \xi_{I_{L}}\left(xyx^{-1}\right). \end{array}$$

From (35) and (38), we get *ζ*_*I*_*L*__(*xyx*^−1^)=*ζ*_*I*_*L*__(*y*). Similarly, from (36) and (39), we get *ξ*_*I*_*L*__(*xyx*^−1^)=*ξ*_*I*_*L*__(*y*). Thus, *I*_*L*_ is LIFNSG-3 of *G*. Hence proved.

**Theorem 1 .**

Let *G* be a finite group and *I*_*L*_=(*G*, *L*, *ζ*_*I*_*L*__, *ξ*_*I*_*L*__, *℘*) be a LIFSG-3 of *G*, such that all (*t*, *s*)-cut subgroups of *I*_*L*_ are normal in *G*. Then, *I*_*L*_ is a LIFNSG-3.

**Proof**

As *G* is finite this implies that Im*ζ*_*I*_*L*__ and Im *ξ*_*I*_*L*__ are finite sets. Suppose Im *ζ*_*I*_*L*__={*t*_1_, *t*_2_,…, *t*_*k*_} with *t*_1_ < *t*_2_,…, <*t*_*k*_ and Im *ξ*_*I*_*L*__={*s*_1_, *s*_2_,…, *s*_*m*_} with *s*_1_ > *s*_2_,…, >*s*_*m*−1_ > *s*_*m*_. Consider *𝒳*={*I*_*L*_^(*t*_*i*_, *s*_*j*_)^ : 1 ≤ *i* ≤ *k*, 1 ≤ *j* ≤ *m*} be the complete set of (*t*_*i*_, *s*_*j*_)-cut subgroup of *I*_*L*_. As each $I_{L}^{\left(t_{i},s_{j}\right)}\underline{⊲}G$, so

(40) $$\begin{array}{c} \frac{I_{L}^{\left(t_{i},s_{j}\right)}}{I_{L}^{\left(t_{i-1},s_{j+1}\right)}}=\left\{x\in G:\zeta_{I_{L}}\left(x\right)=t_{i},\xi_{I_{L}}\left(x\right)=s_{j}\right\}. \end{array}$$

It is normal in *G*, it can be expressed as union of *𝒞*={*xyx*^−1^ : *x* ∈ *G*} and *y* ∈ *I*_*L*_^(*t*_*i*_, *s*_*j*_)^/*I*_*L*_^(*t*_*i*−1_, *s*_*j*+1_)^. Due to normality of cut subgroups, *ζ*_*I*_*L*__(*xyx*^−1^)=*ζ*_*I*_*L*__(*y*) and *ξ*_*I*_*L*__(*xyx*^−1^)=*ξ*_*I*_*L*__(*y*) for all *x*  ∈  *G* and *y* ∈ *I*_*L*_^(*t*_*i*_, *s*_*j*_)^/*I*_*L*_^(*t*_*i*−1_, *s*_*j*+1_)^. We get similar result from each cut-subgroup. Hence, *ζ*_*I*_*L*__(*xyx*^−1^)=*ζ*_*I*_*L*__(*y*) and *ξ*_*I*_*L*__(*xyx*^−1^)=*ξ*_*I*_*L*__(*y*)∀ *x*, *y* ∈ *G*. This implies that *I*_*L*_ is a LIFNSG-3 of *G*.

## 5. Coset and Homomorphism in Lattice-Valued Intuitionistic Fuzzy Subgroup Type-3

In the first section, we introduced the fundamental concepts of the factor group and group homomorphism. In this section, we will discuss these two important features of classical group theory for LIFSG-3.

**Definition 6 .**

For a group *G*, lattice *L* with top and bottom element *T* and *B*. Consider the lattice homomorphism *℘* : *L*⟶[0,1]. Let *I*_*L*_=(*G*, *L*, *ζ*_*I*_*L*__, *ξ*_*I*_*L*__, *℘*) be a LIFSG-3 of *G*. For *x*  ∈  *G* define two maps

(41) $$\begin{array}{c} \zeta_{I_{L}}^{x}:G⟶L\text{ as }\zeta_{I_{L}}^{x}\left(g\right)=\zeta_{I_{L}}\left(gx^{-1}\right)\forall g\in G,\\ \xi_{I_{L}}^{x}:G⟶L\text{ as }\xi_{I_{L}}^{x}\left(g\right)=\xi_{I_{L}}\left(gx^{-1}\right)\forall g\in G. \end{array}$$

Then,

(42) $$\begin{array}{c} ℘\left(\zeta_{I_{L}}^{x}\left(g\right)\right)+℘\left(\xi_{I_{L}}^{x}\left(g\right)\right)=℘\left(\zeta_{I_{L}}\left(gx^{-1}\right)\right)+℘\left(\xi_{I_{L}}\left(gx^{-1}\right)\right)\leq 1. \end{array}$$

This implies that *I*_*L*_^*x*^=(*G*, *L*, *ζ*_*I*_*L*__^*x*^, *ξ*_*I*_*L*__^*x*^, *℘*) is a LIFS-3 of *G*. The LIFS-3

(43) $$\begin{array}{c} I_{L}^{x}=\left(G,L,\zeta_{I_{L}}^{x},\xi_{I_{L}}^{x},℘\right), \end{array}$$

where *G* is called the lattice-valued intuitionistic fuzzy coset type-3 (LIFC-3) of *G* induced by *x* and *I*_*L*_.

**Proposition 7 .**

If *I*_*L*_ is a LIFNSG-3 of *G*, then for any *x*  ∈  *G*,

(44) $$\begin{array}{c} \zeta_{I_{L}}^{x}\left(xg\right)=\zeta_{I_{L}}^{x}\left(gx\right)=\zeta_{I_{L}}\left(g\right),\\ \xi_{I_{L}}^{x}\left(xg\right)=\xi_{I_{L}}^{x}\left(gx\right)\\ =\xi_{I_{L}}\left(g\right)\forall g\in G. \end{array}$$

**Proof**

Suppose *I*_*L*_ is a LIFNSG-3 of *G*. Then, for *x* in *G*

(45) $$\begin{array}{c} \zeta_{I_{L}}^{x}\left(xg\right)=\zeta_{I_{L}}\left(xgx^{-1}\right)\\ =\zeta_{I_{L}}\left(g\right),\\ \zeta_{I_{L}}^{x}\left(gx\right)=\zeta_{I_{L}}\left(gxx^{-1}\right)\\ =\zeta_{I_{L}}\left(g\right). \end{array}$$

Similarly, we proved for *ξ*_*I*_*L*__. Hence proved.

**Theorem 2 .**

Let *I*_*L*_=(*G*, *L*, *ζ*_*I*_*L*__, *ξ*_*I*_*L*__, *℘*) be a LIFNSG-3 of *G*. Let *G*/*I*_*L*_={*I*_*L*_^*x*^ : *x* ∈ *G*} be the collection of all LIFC-3 of *G* induced by *x*  ∈  *G* and *I*_*L*_. Then, *G*/*I*_*L*_ is a group under the binary operation *I*_*L*_^*x*^*oI*_*L*_^*y*^=(*G*, *L*, *ζ*_*I*_*L*__^*xy*^, *ξ*_*I*_*L*__^*xy*^, *℘*) and $\bar{I_{L}}=\left(G/I_{L},L,\bar{\zeta_{I_{L}}},\bar{\xi_{I_{L}}},℘\right)$ is a LIFSG-3 of *G*/*I*_*L*_, where

(46) $$\begin{array}{c} \bar{\zeta_{I_{L}}}\left(I_{L}^{x}\right)=\zeta_{I_{L}}\left(x\right),\\ \bar{\xi_{I_{L}}}\left(I_{L}^{x}\right)=\xi_{I_{L}}\left(x\right)\forall I_{L}^{x}\in G/I_{L}. \end{array}$$

**Proof**

Let *I*_*L*_^*x*_1_^, *I*_*L*_^*x*_2_^, *I*_*L*_^*y*_1_^, *I*_*L*_^*y*_2_^ ∈ *G*/*I*_*L*_ such that *I*_*L*_^*x*_1_^=*I*_*L*_^*y*_1_^ and *I*_*L*_^*x*_2_^=*I*_*L*_^*y*_2_^

Consider *ζ*_*I*_*L*__^*x*_1_*x*_2_^(*g*)=*ζ*_*I*_*L*__(*g*(*x*_1_*x*_2_)^−1^) implies that *ζ*_*I*_*L*__^*x*_1_*x*_2_^(*g*) ≥ *ζ*_*I*_*L*__(*gy*_2_^−1^*y*_1_^−1^)∧*ζ*_*I*_*L*__(*y*_1_*y*_2_*x*_2_^−1^*x*_1_^−1^). As *ζ*_*I*_*L*__^*x*_1_^ = *ζ*_*I*_*L*__^*y*_1_^ and *ζ*_*I*_*L*__^*x*_2_^ = *ζ*_*I*_*L*__^*y*_2_^

(47) $$\begin{array}{c} \Rightarrow \zeta_{I_{L}}\left(gx_{1}^{-1}\right)=\zeta_{I_{L}}\left(gy_{1}^{-1}\right),\\ \Rightarrow \zeta_{I_{L}}\left(gx_{2}^{-1}\right)=\zeta_{I_{L}}\left(gy_{2}^{-1}\right)\forall g\in G. \end{array}$$

If *g* = *y*_1_*y*_2_*x*_2_^−1^, then *ζ*_*I*_*L*__(*y*_1_*y*_2_*x*_2_^−1^*x*_1_^−1^)=*ζ*_*I*_*L*__(*y*_2_*x*_2_^−1^), because *I*_*L*_ is a lattice-valued intuitionistic fuzzy normal subgroup type-3. As *ζ*_*I*_*L*__(*gx*_2_^−1^)=*ζ*_*I*_*L*__(*gy*_2_^−1^), implies that *ζ*_*I*_*L*__(*y*_1_*y*_2_*x*_2_^−1^*x*_1_^−1^)=*ζ*_*I*_*L*__(*e*). Thus, we get that

(48) $$\begin{array}{c} \zeta_{I_{L}}^{x_{1}x_{2}}\left(g\right)\geq \zeta_{I_{L}}^{y_{1}y_{2}}\left(g\right). \end{array}$$

Similarly, we get

(49) $$\begin{array}{c} \xi_{I_{L}}^{x_{1}x_{2}}\left(g\right)=\xi_{I_{L}}^{y_{1}y_{2}}\left(g\right). \end{array}$$

Now we have, *℘*(*ζ*_*I*_*L*__^*x*_1_*x*_2_^(*g*))+*℘*(*ξ*_*I*_*L*__^*x*_1_*x*_2_^(*g*))=*℘*(*ζ*_*I*_*L*__(*g*(*x*_1_*x*_2_)^−1^))+*℘*(*ξ*_*I*_*L*__(*g*(*x*_1_*x*_2_)^−1^))

(50) $$\begin{array}{c} ℘\left(\zeta_{I_{L}}^{x_{1}x_{2}}\left(g\right)\right)+℘\left(\xi_{I_{L}}^{x_{1}x_{2}}\left(g\right)\right)\leq 1. \end{array}$$

Thus, the binary operation is well defined. The associativity of composition of functions implies that the given binary operation is associative. Consider

(51) $$\begin{array}{c} I_{L}^{e}=\left(G,L,\zeta_{I_{L}}^{e},\xi_{I_{L}}^{e},℘\right), \end{array}$$

where *ζ*_*I*_*L*__^*e*^(*g*)=*ζ*_*I*_*L*__(*ge*^−1^)=*ζ*_*I*_*L*__(*g*) and *ξ*_*I*_*L*__^*e*^(*g*)=*ξ*_*I*_*L*__(*ge*^−1^)=*ξ*_*I*_*L*__(*g*)∀ *g* ∈ *G*, this implies that *I*_*L*_^*e*^ = *I*_*L*_ and for any *I*_*L*_^*x*^ ∈ *G*/*I*_*L*_.

(52) $$\begin{array}{c} I_{L}^{e}oI_{L}^{x}=\left(G,L,\zeta_{I_{L}}^{x},\xi_{I_{L}}^{x},℘\right)=I_{L}^{x}. \end{array}$$

Similarly, *I*_*L*_^*x*^ *o* *I*_*L*_^*e*^ = *I*_*L*_^*x*^ this implies that *I*_*L*_^*e*^ is the identity element in *G*/*I*_*L*_. Let *I*_*L*_^*x*^ ∈ *G*/*I*_*L*_, where *x*  ∈  *G*. As *G* is a group so *x*^−1^  ∈  *G*, implies that *I*_*L*_^*x*^−1^^ ∈ *G*/*I*_*L*_ and

(53) $$\begin{array}{c} I_{L}^{x}oI_{L}^{x^{-1}}=\left(G,L,\zeta_{I_{L}}^{xx^{-1}},\xi_{I_{L}}^{xx^{-1}},℘\right),\\ I_{L}^{x^{-1}}oI_{L}^{x}=I_{L}^{e}. \end{array}$$

⇒*G*/*I*_*L*_ is a group.

Consider $\bar{I_{L}}=\left(G/I_{L},L,\bar{\zeta_{I_{L}}},\bar{\xi_{I_{L}}},℘\right)$, where

(54) $$\begin{array}{c} \bar{\zeta_{I_{L}}}\left(I_{L}^{x}\right)=\zeta_{I_{L}}\left(x\right),\\ \bar{\xi_{I_{L}}}\left(I_{L}^{x}\right)=\xi_{I_{L}}\left(x\right)\forall I_{L}^{x}\in \frac{G}{I_{L}},\\ \bar{\zeta_{I_{L}}}\left(I_{L}^{x}oI_{L}^{y}\right)=\bar{\zeta_{I_{L}}}\left(I_{L}^{xy}\right)\\ \bar{\zeta_{I_{L}}}\left(I_{L}^{x}oI_{L}^{y}\right)\geq \bar{\zeta_{I_{L}}}\left(I_{L}^{x}\right)\wedge \bar{\zeta_{I_{L}}}\left(I_{L}^{y}\right),\\ \bar{\zeta_{I_{L}}}\left(I_{L}^{x}oI_{L}^{y}\right)=\bar{\zeta_{I_{L}}}\left(I_{L}^{xy}\right),\\ \bar{\zeta_{I_{L}}}\left(I_{L}^{x}oI_{L}^{y}\right)\geq \bar{\zeta_{I_{L}}}\left(I_{L}^{x}\right)\wedge \bar{\zeta_{I_{L}}}\left(I_{L}^{y}\right),\\ \bar{\xi_{I_{L}}}\left(I_{L}^{x}oI_{L}^{y}\right)=\bar{\xi_{I_{L}}}\left(I_{L}^{xy}\right),\\ \bar{\xi_{I_{L}}}\left(\left(I_{L}^{x}oI_{L}^{y}\right)\right)\leq \bar{\xi_{I_{L}}}\left(I_{L}^{x}\right)\vee \bar{\xi_{I_{L}}}\left(I_{L}^{y}\right),=℘\left(\zeta_{I_{L}}\left(x\right)\right)+℘\left(\left(\xi_{I_{L}}\left(x\right)\right)\right.\leq 1\\ ℘\left(\bar{\zeta_{I_{L}}}\left(I_{L}^{x}\right)\right)+℘\left(\bar{\xi_{I_{L}}}\left(I_{L}^{x}\right)\right)\leq 1\\ ℘\left(\bar{\zeta_{I_{L}}}\left(I_{L}^{x}\right)\right)+℘\left(\bar{\xi_{I_{L}}}\left(I_{L}^{x}\right)\right), \end{array}$$

$\Rightarrow \;\bar{I_{L}}$ is a LIFSG-3 of *G*/*I*_*L*_. Hence proved.

**Definition 7 .**

Let *I*_*L*_ be a LIFNSG-3 of *G*. Then, $\bar{I_{L}}$ is called a lattice intuitionistic fuzzy quotient subgroup type-3 of *G* denoted by LIFQSG − 3.

**Proposition 8 .**

Let *G* be a group and *I*_*L*_ be a LIFNSG-3 of *G*. The map *φ* : *G*⟶*G*/*I*_*L*_ defined by *φ*(*x*)=*I*_*L*_^*x*^∀ *xnG* is epimorphism with

(55) $$\begin{array}{c} \text{ker}\left(\varphi \right)=G_{\zeta_{I_{L}}\xi_{I_{L}}}=\left\{x\in G:\zeta_{I_{L}}\left(e\right)=\zeta_{I_{L}}\left(x\right),\xi_{I_{L}}\left(e\right)=\xi_{I_{L}}\left(x\right)\right\}. \end{array}$$

**Proof**

Let *x*, *y*  ∈  *G*, then *φ*(*xy*)=*I*_*L*_^*xy*^=*I*_*L*_^*x*^*oI*_*L*_^*y*^=*φ*(*x*)*oφ*(*y*) this implies that *φ* is a group homomorphism. Clearly, *φ* is onto,

(56) $$\begin{array}{c} \text{ker}\left(\varphi \right)=\left\{x\in G:\zeta_{I_{L}}\left(e\right)=\zeta_{I_{L}}\left(x\right),\xi_{I_{L}}\left(e\right)=\xi_{I_{L}}\left(x\right)\right\},\\ \text{ker}\left(\varphi \right)=G_{\zeta_{I_{L}}\xi_{I_{L}}}. \end{array}$$

From 1^*st*^ isomorphism theorem we get that *G*/*G*_*ζ*_*I*_*L*__*ξ*_*I*_*L*___≅*G*/*I*_*L*_. Hence proved.

## 6. Group Homomorphism and Lattice-Valued Intuitionistic Fuzzy Subgroup Type-3

**Proposition 9 .**

Let *G* be a group and *I*_*L*_=(*G*, *L*, *ζ*_*I*_*L*__, *ξ*_*I*_*L*__, *℘*) be a lattice-valued intuitionistic fuzzy subgroup type-3 of *G*. Suppose $\vartheta \;:\;G\;⟶\;\overset{´}{G}$ be a group isomorphism. Then, $J_{L}=\left(\overset{´}{G},L,\vartheta o\zeta_{I_{L}},\vartheta o\xi_{I_{L}},℘\right)$, where

(57) $$\begin{array}{c} \left(\vartheta o\zeta_{I_{L}}\right)\left(\overset{´}{g}\right)=\zeta_{I_{L}}\left(\vartheta^{-1}\left(\overset{´}{g}\right)\right),\\ \left(\vartheta o\xi_{I_{L}}\right)\left(\overset{´}{g}\right)=\xi_{I_{L}}\left(\vartheta^{-1}\left(\overset{´}{g}\right)\right), \end{array}$$

is a LIFSG-3 of $\overset{´}{G}$.

**Proof**

Let ${\overset{´}{g}}_{1},{\overset{´}{g}}_{2}\;\in \;\overset{´}{G}$. Then,

(58) $$\begin{array}{c} \left(\vartheta o\zeta_{I_{L}}\right)\left({\overset{´}{g}}_{1}{\overset{´}{g}}_{2}\right)=\zeta_{I_{L}}\left(\vartheta^{-1}\left({\overset{´}{g}}_{1}{\overset{´}{g}}_{2}\right)\right),\\ \left(\vartheta o\zeta_{I_{L}}\right)\left({\overset{´}{g}}_{1}{\overset{´}{g}}_{2}\right)\geq \vartheta o\zeta_{I_{L}}\left({\overset{´}{g}}_{1}\right)\wedge \vartheta o\zeta_{I_{L}}\left({\overset{´}{g}}_{2}\right). \end{array}$$

Similarly, $\left(\vartheta o\xi_{I_{L}}\right)\left({\overset{´}{g}}_{1}{\overset{´}{g}}_{2}\right)\leq \vartheta o\xi_{I_{L}}\left({\overset{´}{g}}_{1}\right)\vee \vartheta o\xi_{I_{L}}\left({\overset{´}{g}}_{2}\right)$. Let $a\;\in \;\overset{´}{G}$. Then,

(59) $$\begin{array}{c} \left(\vartheta o\zeta_{I_{L}}\right)\left(a^{-1}\right)=\zeta_{I_{L}}\left(\vartheta^{-1}\left(a^{-1}\right)\right),\\ \left(\vartheta o\zeta_{I_{L}}\right)\left(a^{-1}\right)=\vartheta o\zeta_{I_{L}}\left(a\right). \end{array}$$

Similarly, (*ϑoξ*_*I*_*L*__)(*a*^−1^)=*ϑoξ*_*I*_*L*__(*a*). Now,

(60) $$\begin{array}{c} ℘\left(\vartheta o\zeta_{I_{L}}\left(\overset{´}{g}\right)\right)+℘\left(\vartheta o\xi_{I_{L}}\left(\overset{´}{g}\right)\right)=℘\left(\zeta_{I_{L}}\left(\vartheta^{-1}\left(\overset{´}{g}\right)\right)\right)+℘\left(\xi_{I_{L}}\left(\vartheta^{-1}\left(\overset{´}{g}\right)\right)\right)\leq 1\\ ℘\left(\vartheta o\zeta_{I_{L}}\left(\overset{´}{g}\right)\right)+℘\left(\vartheta o\xi_{I_{L}}\left(\overset{´}{g}\right)\right)\leq 1.\frac{1}{2}. \end{array}$$

⇒ *J*_*L*_ is a LIFSG-3 of $\overset{´}{G}$. Hence proved.

Similar to the above proposition, now we will relate LIFNSG-3 of *G* and $\overset{´}{G}$.

**Proposition 10 .**

Let *G* be a group and *I*_*L*_=(*G*, *L*, *ζ*_*I*_*L*__, *ξ*_*I*_*L*__, *℘*) be a LIFNSG-3 of *G*. If $\vartheta \;:\;G\;⟶\;\overset{´}{G}$ be a group isomorphism, then $J_{L}=\left(\overset{´}{G},L,\vartheta o\zeta_{I_{L}},\vartheta o\xi_{I_{L}},℘\right)$ is a LIFNSG-3 of $\overset{´}{G}$.

**Proof**

Suppose *I*_*L*_ is a LIFNSG-3 of *G*. Let $u,v\;\in \;\overset{´}{G}$. Then, for $u,v\in \overset{´}{G}$

(61) $$\begin{array}{c} \vartheta o\zeta_{I_{L}}\left(uvu^{-1}\right)=\zeta_{I_{L}}\left(\vartheta^{-1}\left(uvu^{-1}\right)\right)\\ =\zeta_{I_{L}}\left(\vartheta^{-1}\left(v\right)\right.\\ =\vartheta o\zeta_{I_{L}}\left(v\right). \end{array}$$

Similarly, *ϑoξ*_*I*_*L*__(*uvu*^−1^)=*ξ*_*I*_*L*__(*ϑ*^−1^(*v*))=*ϑoξ*_*I*_*L*__(*v*)⇒*J*_*L*_ is a LIFNSG-3 of $\overset{´}{G}$. Hence proved.

In the following proposition we will induce LIFSG-3 of *G* from the LIFS-3 of $\overset{´}{G}$.

**Proposition 11 .**

Let $G,\overset{´}{G}$ be two groups and $\varphi \;:\;G\;⟶\;\overset{´}{G}$ be a group homomorphism. Suppose $J_{L}=\left(\overset{´}{G},L,\zeta_{J_{L}},\xi_{J_{L}},℘\right)$ be a LIFSG-3 of $\overset{´}{G}$. Then, *I*_*L*_=(*G*, *L*, *ζ*_*J*_*L*__*oφ*, *ξ*_*J*_*L*__*oφ*, *℘*) is a LIFSG-3 of *G*. Where ∀ *g* ∈ *G*

(62) $$\begin{array}{c} \zeta_{J_{L}}\;o\;\varphi \left(g\right)=\;\zeta_{J_{L}}\left(\varphi \left(g\right)\right),\\ \xi_{J_{L}}\;o\;\varphi \left(g\right)\;=\;\xi_{J_{L}}\left(\varphi \left(g\right)\right). \end{array}$$

**Proof**

Let *g*_1_, *g*_2_  ∈  *G*. Then,

(63) $$\begin{array}{c} \zeta_{J_{L}}o\;\varphi \left(g_{1}g_{2}\right)=\zeta_{J_{L}}\left(\varphi \left(g_{2}g_{2}\right)\right),\\ \zeta_{J_{L}}o\;\varphi \left(g_{1}g_{2}\right)\geq \zeta_{J_{L}}o\;\varphi \left(g_{2}\right)\wedge \zeta_{J_{L}}o\varphi \left(g_{2}\right). \end{array}$$

Similarly, *ξ*_*J*_*L*__*o* *φ*(*g*_1_*g*_2_) ≤ *ξ*_*J*_*L*__*o* *φ*(*g*_2_)∨*ξ*_*J*_*L*__*o* *φ*(*g*_2_). Now,

(64) $$\begin{array}{c} \zeta_{J_{L}}o\;\varphi \left(g^{-1}\right)=\zeta_{J_{L}}\left(\varphi \left(g^{-1}\right)\right),\\ \zeta_{J_{L}}o\;\varphi \left(g^{-1}\right)=\zeta_{J_{L}}\left(\varphi \left(g\right)\right),\\ \zeta_{J_{L}}o\;\varphi \left(g^{-1}\right)=\zeta_{J_{L}}o\;\varphi \left(g\right). \end{array}$$

Similarly, *ξ*_*J*_*L*__*o* *φ*(*g*^−1^)=*ξ*_*J*_*L*__*o* *φ*(*g*).

(65) $$\begin{array}{c} ℘\left(\zeta_{J_{L}}o\varphi \left(g\right)\right)+℘\left(\xi_{J_{L}}o\varphi \left(g\right)\right)=℘\left(\zeta_{J_{L}}\left(\varphi \left(g\right)\right)\right)+℘\left(\xi_{J_{L}}\left(\varphi \left(g\right)\right)\right)\leq 1\\ ℘\left(\zeta_{J_{L}}o\varphi \left(g\right)\right)+℘\left(\xi_{J_{L}}o\varphi \left(g\right)\right)\leq 1. \end{array}$$

⇒(*G*, *L*, *ζ*_*J*_*L*__*oφ*, *ξ*_*J*_*L*__*oφ*, *℘*) is a LIFSG-3 of *G*. Hence proved.

**Proposition 12 .**

Let *G* and $\overset{´}{G}$ be two groups, $\varphi \;:\;G\;⟶\;\overset{´}{G}$ be a group homomorphism and

(66) $$\begin{array}{c} J_{L}=\left(\overset{´}{G},L,\zeta_{J_{L}},\xi_{J_{L}},℘\right), \end{array}$$

be a LIFNSG-3 of $\overset{´}{G}$. Then,

(67) $$\begin{array}{c} I_{L}=\left(G,L,\zeta_{J_{L}}o\varphi ,\xi_{J_{L}}o\varphi ,℘\right), \end{array}$$

is a LIFNSG-3 of *G*.

**Proof**

Let *u*, *v*  ∈  *G*. Then,

(68) $$\begin{array}{c} \zeta_{J_{L}}o\varphi \left(uvu^{-1}\right)=\zeta_{J_{L}}\left(\varphi \left(uvu^{-1}\right)\right),\\ =\zeta_{J_{L}}\left(\varphi \left(u\right)\varphi \left(v\right)\varphi \left(u^{-1}\right)\right),\\ =\zeta_{J_{L}}\left(\varphi \left(v\right)\right),\\ =\zeta_{J_{L}}o\varphi \left(v\right). \end{array}$$

Similarly, *ξ*_*J*_*L*__*oφ*(*uvu*^−1^)=*ξ*_*J*_*L*__*oφ*(*v*). ⇒ *I*_*L*_ is a LIFNSG-3 of *G*. Hence proved.

**Theorem 3 .**

Let *G* and $\overset{´}{G}$ be any two groups and $\vartheta \;:\;G\;⟶\;\overset{´}{G}$ be a group isomorphism. Let *L* be a lattice with top element *T* and bottom element *B* and *℘* : *L*⟶[0,1] be a lattice homomorphism. Let *I*_*G*_^LIFSG−3^ and $I_{\overset{´}{G}}^{\text{LIFSG}-3}$ be the collections of LIFSG-3 of *G* and $\overset{´}{G}$, respectively. Then, the map

(69) $$\begin{array}{c} \hat{\vartheta }:I_{G}^{\text{LIFSG}-3}⟶I_{\overset{´}{G}}^{\text{LIFSG}-3} \end{array}$$

Defined by

(70) $$\begin{array}{c} \hat{\vartheta }\left(G,L,\zeta_{I_{L}},\xi_{I_{L}},℘\right)=\left(\overset{´}{\bar{G}},L,\vartheta o\zeta_{I_{L}},\vartheta o\xi_{I_{L}},℘\right), \end{array}$$

is bijective.

**Proof**

From previous propositions we know that if

(71) $$\begin{array}{c} I_{L}=\left(G,L,\zeta_{I_{L}},\xi_{I_{L}},℘\right)\in I_{G}^{\text{LIFSG}-3}. \end{array}$$

Then,

(72) $$\begin{array}{c} J_{L}=\left(\overset{´}{G},L,\vartheta o\zeta_{I_{L}},\vartheta o\xi_{I_{L}},℘\right)\in I_{\overset{´}{G}}^{\text{LIFSG}-3}. \end{array}$$

Now, if

(73) $$\begin{array}{c} \vartheta o\zeta_{I_{L}}^{1}=\vartheta o\zeta_{I_{L}}^{2},\\ \Rightarrow \vartheta o\zeta_{I_{L}}^{1}\left(\overset{´}{g}\right)=\vartheta o\zeta_{I_{L}}^{2}\left(\overset{´}{g}\right)\forall \overset{´}{g}\in \overset{´}{G},\\ \Rightarrow \zeta_{I_{L}}^{1}\left(\vartheta^{-1}\left(\overset{´}{g}\right)\right)=\zeta_{I_{L}}^{2}\left(\vartheta^{-1}\left(\overset{´}{g}\right)\right)\forall \overset{´}{g}\in \overset{´}{G}. \end{array}$$

As *ϑ* is bijective

(74) $$\begin{array}{c} \Rightarrow \left\{\vartheta^{-1}\left(\overset{´}{g}\right):\overset{´}{g}\in \overset{´}{G}\right\}=G\pm ,\\ \Rightarrow \zeta_{I_{L}}^{1}=\zeta_{I_{L}}^{2}. \end{array}$$

Similarly,

(75) $$\begin{array}{c} \vartheta o\xi_{I_{L}}^{1}=\vartheta o\xi_{I_{L}}^{2},\\ \Rightarrow \xi_{I_{L}}^{1}\left(\vartheta^{-1}\left(\overset{´}{g}\right)\right)=\xi_{I_{L}}^{2}\left(\vartheta^{-1}\left(\overset{´}{g}\right)\right)\forall \overset{´}{g}\in \overset{´}{G}. \end{array}$$

As *ϑ* is bijective, this implies *ξ*_*I*_*L*__^1^ = *ξ*_*I*_*L*__^2^. Thus, we get that, if

(76) $$\begin{array}{c} \hat{\vartheta }\left(G,L,\zeta_{I_{L}}^{1},\xi_{I_{L}}^{1},℘\right)=\hat{\vartheta }\left(G,L,\zeta_{I_{L}}^{2},\xi_{I_{L}}^{2},℘\right), \end{array}$$

then $\left(G,L,\zeta_{I_{L}}^{1},\xi_{I_{L}}^{1},℘\right)=\left(G,L,\zeta_{I_{L}}^{2},\xi_{I_{L}}^{2},℘\right)\Rightarrow \hat{\vartheta }$ is injective. Now, if

(77) $$\begin{array}{c} \left(\overset{´}{G},L,\zeta_{J_{L}},\xi_{J_{L}},℘\right)\in I_{\overset{´}{G}}^{\text{LIFSG}-3}, \end{array}$$

then (*G*, *L*, *ζ*_*J*_*L*__*oϑ*, *ξ*_*J*_*L*__*oϑ*, *℘*) ∈ *I*_*G*_^LIFSG−3^ and $\forall \overset{´}{g}\in \overset{´}{G}$,

(78) $$\begin{array}{c} \left(\vartheta o\left(\zeta_{J_{L}}o\vartheta \right)\right)\left(\overset{´}{g}\right)=\left(\zeta_{J_{L}}o\vartheta \right)\left(\vartheta^{-1}\left(\overset{´}{g}\right)\right)=\zeta_{J_{L}}\left(\overset{´}{g}\right),\\ \Rightarrow \vartheta o\left(\zeta_{J_{L}}o\vartheta \right)=\zeta_{J_{L}}. \end{array}$$

Similarly, *ϑo*(*ξ*_*J*_*L*__*o* *ϑ*)=*ξ*_*J*_*L*__. This implies that $\hat{\vartheta }$ is bijective. Hence proved.

**Theorem 4 .**

Let *G* be a group, *L* and $\overset{´}{L}$ be any two lattices with top elements *T* and $\overset{´}{T}$, and bottom elements *B* and $\overset{´}{B}$, $f\;:\;L\;⟶\;\overset{´}{L}$ and *℘* : *L*⟶[0,1] be lattice homomorphisms such that $f\left(T\right)=\overset{´}{T}$, $f\left(B\right)=\overset{´}{B}$, and *℘*(*T*)=1,  *℘*(*B*)=0. If *I*_*L*_=(*G*, *L*, *ζ*_*I*_*L*__, *ξ*_*I*_*L*__, *℘*) be a LIFSG-3 of *G*, then $J_{\overset{´}{L}}=\left(G,\overset{´}{L},fo\;\zeta_{I_{L}},fo\;\xi_{I_{L}},\overset{´}{℘}\right)$ is also a LIFSG-3 of *G*. Where

(79) $$\begin{array}{c} fo\zeta_{I_{L}},fo\xi_{I_{L}}:G⟶L⟶\overset{´}{L}, \end{array}$$

are defined as

(80) $$\begin{array}{c} fo\zeta_{I_{L}}\left(g\right)=f\left(\zeta_{I_{L}}\left(g\right)\right),\\ fo\xi_{I_{L}}\left(g\right)=f\left(\xi_{I_{L}}\left(g\right)\right), \end{array}$$

and

(81) $$\begin{array}{c} \overset{´}{℘}\left(\overset{´}{l}\right)=\underset{f\left(l\right)=\overset{´}{l}}{\text{sup}}\left(℘\left(f^{-1}\left(\overset{´}{l}\right)\right)\right). \end{array}$$

**Proof**

Let *g*_1_, *g*_2_  ∈  *G*. Then,

(82) $$\begin{array}{c} fo\zeta_{I_{L}}\left(g_{1}g_{2}\right)=f\left(\zeta_{I_{L}}\left(g_{1}g_{2}\right)\right)\geq f\left(\zeta_{I_{L}}\left(g_{1}\right)\wedge \zeta_{I_{L}}\left(g_{2}\right)\right)\geq f\left(\zeta_{I_{L}}\left(g_{1}\right)\right)\wedge f\\ \left(\zeta_{I_{L}}\left(g_{2}\right)\right)\geq fo\zeta_{I_{L}}\left(g_{1}\right)\wedge fo\zeta_{I_{L}}\left(g_{2}\right). \end{array}$$

Similarly, *foξ*_*I*_*L*__(*g*_1_*g*_2_) ≤ *foξ*_*I*_*L*__(*g*_1_)∨*foξ*_*I*_*L*__(*g*_2_). Let *g* ∈ *G* and

(83) $$\begin{array}{c} \overset{´}{℘}\left(fo\zeta_{I_{L}}\left(g\right)\right)=\text{sup}\left(℘\left(f^{-1}\left(fo\zeta_{I_{L}}\left(\left(g\right)\right)\right)\right)\right),\\ =M_{1}\frac{1}{2},\\ \overset{´}{℘}\left(\left(fo\xi_{I_{L}}\left(g\right)\right)\right)=\text{sup}\left(℘\left(f^{-1}\left(fo\xi_{I_{L}}\left(g\right)\right)\right)\right),\\ =M_{2}. \end{array}$$

Then, *M*_1_ and *M*_2_ are of the form

(84) $$\begin{array}{c} M_{1}=℘\left(f^{-1}\left(fo\zeta_{I_{L}}\left(g\right)\right)\right)=℘\left(\zeta_{I_{L}}\left(g\right)\right),\\ M_{2}=℘\left(f^{-1}\left(fo\xi_{I_{L}}\left(g\right)\right)\right)=℘\left(\xi_{I_{L}}\left(g\right)\right). \end{array}$$

Thus,

(85) $$\begin{array}{c} \overset{´}{℘}\left(fo\zeta_{I_{L}}\left(g\right)\right)+\overset{´}{℘}\left(fo\xi_{I_{L}}\left(g\right)\right)=℘\left(\zeta_{I_{L}}\left(g\right)\right)+℘\left(\xi_{I_{L}}\left(g\right)\right)\leq 1. \end{array}$$

Hence, we get the required result.

**Theorem 5 .**

Let *G* and $\overset{´}{G}$ be any two group and $\vartheta \;:\;G\;⟶\;\overset{´}{G}$ be a group isomorphism. Let *L* and $\overset{´}{L}$ be any two lattice with top elements *T* and $\overset{´}{T}$, and bottom elements *B* and $\overset{´}{B}$. Let $f\;:\;L\;⟶\;\overset{´}{L}$ and *℘* : *L*⟶[0,1] be lattice homomorphisms such that $f\left(T\right)=\overset{´}{T}$, $f\left(B\right)\;=\;\overset{´}{B}$, *℘*(*T*)=1, *℘*(*B*)=0. Let *I*_*L*_=(*G*, *L*, *ζ*_*I*_*L*__, *ξ*_*I*_*L*__, *℘*) be a LIFSG-3 of *G*. Then,

(86) $$\begin{array}{c} J_{\overset{´}{L}}=\left(\overset{´}{G},\overset{´}{L},\zeta_{J_{\overset{´}{L}}},\xi_{J_{\overset{´}{L}}},\overset{´}{℘}\right), \end{array}$$

is a LIFSG-3 of $\overset{´}{G}$, where

(87) $$\begin{array}{c} \zeta_{J_{\overset{´}{L}}}=\vartheta o\left(fo\zeta_{I_{L}}\right),\\ \;\xi_{J_{\overset{´}{L}}}=\vartheta o\left(fo\xi_{I_{L}}\right),\\ \overset{´}{℘}\left(\overset{´}{l}\right)=\underset{f\left(l\right)=\overset{´}{l}}{\text{sup}}\left(℘\left(f^{-1}\left(\overset{´}{l}\right)\right)\right). \end{array}$$

**Proof**

If *I*_*L*_=(*G*, *L*, *ζ*_*I*_*L*__, *ξ*_*I*_*L*__, *℘*) is a LIFSG-3 of *G*, then from Theorem 4

(88) $$\begin{array}{c} {\overset{´}{B}}_{L}=\left(G,\overset{´}{L},fo\zeta_{I_{L}},fo\xi_{I_{L}},\overset{´}{℘}\right), \end{array}$$

is a LIFSG-3 of *G*. From Proposition 9

(89) $$\begin{array}{c} {\overset{´}{C}}_{L}=\left(\overset{´}{G},\overset{´}{L},\vartheta o\left(fo\zeta_{I_{L}}\right),\vartheta o\left(fo\xi_{I_{L}}\right),\overset{´}{℘}\right), \end{array}$$

is a LIFSG-3 of $\overset{´}{G}$.

**Theorem 6 .**

For a group *G*, lattice *L* with top and bottom elements *T* and *B*, respectively, lattice homomorphism *℘* : *L*⟶[0,1]. Let *I*_*L*_=(*G*, *L*, *ζ*_*I*_*L*__, *ξ*_*I*_*L*__, *℘*) be a LIFNSG-3 of *G*. Then, the set

(90) $$\begin{array}{c} G_{\zeta_{I_{L}}\xi_{I_{L}}}=\left\{x\in G:\zeta_{I_{L}}\left(x\right)=\zeta_{I_{L}}\left(e\right),\xi_{I_{L}}\left(x\right)=\xi_{I_{L}}\left(e\right)\right\}, \end{array}$$

is normal in *G* and

(91) $$\begin{array}{c} \hat{I_{L}}=\left(\frac{G}{G_{\zeta_{I_{L}}\xi_{I_{L}}}},L,\hat{\zeta_{I_{L}}},\hat{\xi_{I_{L}}},℘\right). \end{array}$$

$\left(\text{where}\hat{\zeta_{I_{L}}}\left(xG_{\zeta_{I_{L}}\xi_{I_{L}}}\right)=\zeta_{I_{L}}\left(x\right),\;\hat{\xi_{I_{L}}}\left(xG_{\zeta_{I_{L}}\xi_{I_{L}}}\right)=\xi_{I_{L}}\left(x\right)\text{forall}x\in G\right)$ is a LIFNSG-3 of *G*/*G*_*ζ*_*I*_*L*__*ξ*_*I*_*L*___.

Conversely, if *N* ⊲ *G* and *I*_*L*_^*N*^=(*G*/*N*, *L*, *ζ*_*I*_*L*__^*N*^, *ξ*_*I*_*L*__^*N*^, *℘*) is a LIFNSG-3 of *G*/*N*, then *I*_NL_=(*G*, *L*, *ζ*_*I*_NL__, *ξ*_*I*_NL__, *℘*) is a LIFNSG-3 of *G*, where

(92) $$\begin{array}{c} \zeta_{I_{\text{NL}}}:G⟶L,\;\xi_{I_{\text{NL}}}:G⟶L, \end{array}$$

are defined as

(93) $$\begin{array}{c} \zeta_{I_{NL}}\left(x\right)=\zeta_{I_{NL}}^{N}\left(xN\right),\\ \xi_{I_{NL}}\left(x\right)=\xi_{I_{NL}}^{N}\left(xN\right)\forall x\in G. \end{array}$$

**Proof**

Let *I*_*L*_ be a LIFNSG-3 of *G*. Then, *ζ*_*I*_*L*__(*x*) ≤ *ζ*_*I*_*L*__(*e*) and *ξ*_*I*_*L*__(*x*) ≥ *ξ*_*I*_*L*__(*e*)∀ *x* ∈ *G*, implies that *G*_*ζ*_*I*_*L*__*ξ*_*I*_*L*___ is a cut subgroup so it is normal in *G* and allow to construct factor group *G*/*G*_*ζ*_*I*_*L*__*ξ*_*I*_*L*___. Now, for the map $\hat{\zeta_{I_{L}}}:G/G_{\zeta_{I_{L}}\xi_{I_{L}}}⟶L$

(94) $$\begin{array}{c} \hat{\zeta_{I_{L}}}\left(xG_{\zeta_{I_{L}}\xi_{I_{L}}}.yG_{\zeta_{I_{L}}\xi_{I_{L}}}\right)=\hat{\zeta_{I_{L}}}\left(\left(xy\right)G_{\zeta_{I_{L}}\xi_{I_{L}}}\right),\\ \hat{\zeta_{I_{L}}}\left(xG_{\zeta_{I_{L}}\xi_{I_{L}}}.yG_{\zeta_{I_{L}}\xi_{I_{L}}}\right)=\zeta_{I_{L}}\left(xy\right),\\ \hat{\zeta_{I_{L}}}\left(xG_{\zeta_{I_{L}}\xi_{I_{L}}}.yG_{\zeta_{I_{L}}\xi_{I_{L}}}\right)\geq \zeta_{I_{L}}\left(x\right)\wedge \zeta_{I_{L}}\left(y\right),\\ \hat{\zeta_{I_{L}}}\left(xG_{\zeta_{I_{L}}\xi_{I_{L}}}.yG_{\zeta_{I_{L}}\xi_{I_{L}}}\right)\geq \hat{\zeta_{I_{L}}}\left(xG_{\zeta_{I_{L}}\xi_{I_{L}}}\right)\wedge \hat{\zeta_{I_{L}}}\left(yG_{\zeta_{I_{L}}\xi_{I_{L}}}\right). \end{array}$$

Similarly, for the map $\hat{\xi_{I_{L}}}:G/G_{\zeta_{I_{L}}\xi_{I_{L}}}⟶L$

(95) $$\begin{array}{c} \hat{\xi_{I_{L}}}\left(xG_{\zeta_{I_{L}}\xi_{I_{L}}}.yG_{\zeta_{I_{L}}\xi_{I_{L}}}\right)\leq \hat{\xi_{I_{L}}}\left(xG_{\zeta_{I_{L}}\xi_{I_{L}}}\right)\vee \hat{\xi_{I_{L}}}\left(yG_{\zeta_{I_{L}}\xi_{I_{L}}}\right). \end{array}$$

Also

(96) $$\begin{array}{c} ℘\left(\hat{\zeta_{I_{L}}}\left(xG_{\zeta_{I_{L}}\xi_{I_{L}}}\right)\right)+℘\left(\hat{\xi_{I_{L}}}\left(xG_{\zeta_{I_{L}}\xi_{I_{L}}}\right)\right)=℘\left(\zeta_{I_{L}}\left(x\right)\right)+℘\left(\xi_{I_{L}}\left(x\right)\right)\\ \Rightarrow ℘\left(\hat{\zeta_{I_{L}}}\left(xG_{\zeta_{I_{L}}\xi_{I_{L}}}\right)\right)+℘\left(\hat{\xi_{I_{L}}}\left(xG_{\zeta_{I_{L}}\xi_{I_{L}}}\right)\right)\leq 1. \end{array}$$

$\Rightarrow \;\hat{I_{L}}$ is a LIFNSG-3 of *G*/*G*_*ζ*_*I*_*L*__*ξ*_*I*_*L*___. Conversely, suppose

(97) $$\begin{array}{c} I_{L}^{N}=\left(G/N,L,\zeta_{I_{L}}^{N},\xi_{I_{L}}^{N},℘\right), \end{array}$$

be a LIFNSG-3 of *G*/*N*. Define *ζ*_*I*_NL__ : *G*⟶*L* as

(98) $$\begin{array}{c} \zeta_{I_{NL}}\left(x\right)=\zeta_{I_{L}}^{N}\left(xN\right). \end{array}$$

Then,

(99) $$\begin{array}{c} \zeta_{I_{NL}}\left(xy\right)=\zeta_{I_{L}}^{N}\left(xyN\right),\\ \zeta_{I_{\text{NL}}}\left(xy\right)=\zeta_{I_{L}}^{N}\left(xN.yN\right),\\ \zeta_{I_{\text{NL}}}\left(xy\right)\geq \zeta_{I_{L}}^{N}\left(xN\right)\wedge \zeta_{I_{L}}^{N}\left(yN\right),\\ \zeta_{I_{\text{NL}}}\left(xy\right)\geq \zeta_{I_{\text{NL}}}\left(x\right)\wedge \zeta_{I_{\text{NL}}}\left(y\right). \end{array}$$

Define *ξ*_*I*_NL__ : *G*⟶*L* as

(100) $$\begin{array}{c} \xi_{I_{\text{NL}}}\left(x\right)=\xi_{I_{L}}^{N}\left(xN\right). \end{array}$$

Then,

(101) $$\begin{array}{c} \xi_{I_{\text{NL}}}\left(xy\right)\leq \xi_{I_{\text{NL}}}\left(x\right)\vee \xi_{I_{\text{NL}}}\left(y\right),\\ ℘\left(\zeta_{I_{\text{NL}}}\left(x\right)\right)+℘\left(\xi_{I_{\text{NL}}}\left(x\right)\right)=℘\left(\zeta_{I_{L}}^{N}\left(xN\right)\right)+℘\left(\xi_{I_{L}}^{xN}\right),\\ \Rightarrow ℘\left(\zeta_{I_{\text{NL}}}\left(x\right)\right)+℘\left(\xi_{I_{\text{NL}}}\left(x\right)\right)\leq 1, \end{array}$$

⇒*I*_NL_ is a LIFNSG-3 of *G*.

## 7. Conclusion

The article is about the study of group theocratic concepts in a lattice-valued intuitionistic fuzzy type-3 environment. The structure is established by introducing lattice homomorphism, membership and nonmembership grades obeying certain laws for the binary operation defined on the group and inverses of elements under that operation. It is concluded that the level sets of LIFSG-3 of a group *G* are exactly the subgroups of *G*, and conversely, any LIFS-3 of *G* whose level sets are subgroups of *G* is LIFSG-3. Lattice-valued intuitionistic fuzzy normal subgroups type-3 and lattice-valued intuitionistic fuzzy factor subgroups type-3 of *G* are governed by normal subgroups and factor groups of *G*. Structure preserving LIFSG-3 maps are also discussed, and it is observed that they can be derived by extending group homomorphism. In the future, the notion foundations laid in this article can be used to find the Abelian subgroups of finite *p*-groups [34], verify Lagrange's theorem [35, 36], compute annihilator [37], aggregation [38], and fundamental isomorphism theorems [39] for lattice-valued intuitionistic fuzzy subgroups type-3. There are several generalizations of fuzzy sets [40–42] where lattice-valued algebraic structures can be defined by replacing [0,1] with a suitable lattice *L*. The research findings can be utilized for application in algebra [43, 44] and real-life problems [45] to handle uncertainty and ambiguity more accurately.

## Data Availability

No data were used to support this study.

## References

[1] Zadeh L. A. (1965). Fuzzy sets. *Information and Control*.

[2] Atansassov K. T. (1986). Intuitionistic fuzzy sets. *Fuzzy Sets and Systems*.

[3] Atanassov K. T. (2016). Geometrical interpretation of the elements of the intuitionistic fuzzy objects. *International Journal of Bioautomation*.

[4] Goguen J. A. (1967). L-fuzzy sets. *Journal of Mathematical Analysis and Applications*.

[5] Atanassov K. T., Stoeva S. (1984). Intuitionistic L-fuzzy sets. *Cybernetic and System Research*.

[6] Gerstenkorn T., Tepavĉević A. (2004). Lattice valued intuitionistic fuzzy sets. *Central European Journal of Mathematics*.

[7] Rosenfeld A. (1971). Fuzzy groups. *Journal of Mathematical Analysis and Applications*.

[8] Anthony J. M., Sherwood H. (1979). Fuzzy groups redefined. *Journal of Mathematical Analysis and Applications*.

[9] Anthony J. M., Sherwood H. (1982). A characterization of fuzzy subgroups. *Fuzzy Sets and Systems*.

[10] Bhattacharya P. (1987). Fuzzy subgroups: some characterizations. *Journal of Mathematical Analysis and Applications*.

[11] Das P. S. (1981). Fuzzy groups and level subgroups. *Journal of Mathematical Analysis and Applications*.

[12] Liu J. (1982). Fuzzy invariant subgroups and fuzzy ideals. *Fuzzy Sets and Systems*.

[13] Ajmal N., Prajapati A. S. (1992). Fuzzy cosets and Fuzzy normal subgroups. *Information Sciences*.

[14] Mukherjee N., Bhattacharya P. (1984). Fuzzy normal subgroups and fuzzy cosets. *Information Sciences*.

[15] Mukherjee N. P., Bhattacharya P. (1986). Fuzzy groups: some group-theoretic analogs. *Information Sciences*.

[16] Kumar I. J., Saxena P. K., Yadav P. (1992). Fuzzy normal subgroups and fuzzy quotients. *Fuzzy Sets and Systems*.

[17] Tarnauceanu M. (2015). Classifying fuzzy normal subgroups of finite groups. *Iranian Journal of Fuzzy System*.

[18] Choudhury F. P., Chakraborty A. B., Khare S. S. (1988). A note on fuzzy subgroups and fuzzy homomorphism. *Journal of Mathematical Analysis and Applications*.

[19] Addis G. M. (2018). Fuzzy homomorphism theorems on groups. *Korean Journal of Mathematics*.

[20] Malik D. S., Mordeson J. N., Nair P. S. (1992). Fuzzy normal subgroups in fuzzy subgroups. *Journal of the Korean Mathematical Society*.

[21] Mishref M. A. (1995). Normal fuzzy subgroups and fuzzy normal series of finite groups. *Fuzzy Sets and Systems*.

[22] Zhan G., Zhisong T. (2004). Intuitionistic M-fuzzy groups. *Soochow Journal of Mathematics*.

[23] Li X. P., Gui J. W. (2011). Intuitionistic fuzzy groups. *Hacettepe Journal of Mathematics and Statistics*.

[24] Tarsuslu S., Tarsuslu A., Tarsuslu A., Çitil M. (2018). Intuitionistic fuzzy action of a group on a set. *Notes on Intuitionistic Fuzzy Sets*.

[25] Bal M., Ahmad K. D., Hajjari A. A. (2022). A short note on the kernel subgroup of intuitionistic fuzzy groups. *Journal of Neutrosophic and Fuzzy Systems*.

[26] Negoiţă C. V., Ralescu D. A. (1975). *Applications of Fuzzy Sets to Systems Analysis*.

[27] Riaz A., Kousar S. *Lattice Valued Intuitionistic Fuzzy Type-3 Error-Correcting Reed Muller Codes*.

[28] Robert D., Combescure M. (2021). *Coherent States and Applications in Mathematical Physics*.

[29] Igusa K., Martsinkovsky A., Todorov G. (2021). *Representations of Algebras, Geometry and Physics*.

[30] Bachtis D., Aarts G., Di Renzo F., Lucini B. (2022). Inverse renormalization group in quantum field theory. *Physical Review Letters*.

[31] Bishop D. M. (1993). *Group Theory and Chemistry*.

[32] Mushtaq Q., Mumtaz N. (2018). PSL(2,7)$$PSL\left( 2,7\right) $$ P S L 2 , 7 and carbon allotrope D168 Schwarzite. *Journal of Mathematical Chemistry*.

[33] Sebastian S., Babu Sundar S. (1994). Commutative L-fuzzy subgroups. *Fuzzy Sets and Systems*.

[34] Adebisi S. A., Ogiugo M., Enioluwafe M. (2021). The abelian groups of large order: perspective from (fuzzy) subgroups of finite p-groups. *Mathematics and Computer Science*.

[35] Alolaiyan H., Shuaib U., Latif L., Razaq A. (2019). t-Intuitionistic ftheorem of t-intuitionistic fuzzy subgroup. *IEEE Access*.

[36] Kattan D. A., Amin M., Bariq A. (2022). Certain structure of Lagrange’s theorem with the application of interval-valued intuitionistic fuzzy subgroups. *Journal of Function Spaces*.

[37] Ardanza-Trevijano S., Chasco M. J., Elorza J. (2019). The annihilator of fuzzy subgroups. *Fuzzy Sets and Systems*.

[38] Bejines C., Chasco M. J., Elorza J. (2021). Aggregation of fuzzy subgroups. *Fuzzy Sets and Systems*.

[39] Latif L., Shuaib U., Alolaiyan H., Razaq A. (2018). On fundamental theorems of t-intuitionistic fuzzy isomorphism of t-intuitionistic fuzzy subgroups. *IEEE Access*.

[40] Gégény D., Radeleczki S. (2022). Rough L-fuzzy sets: their representation and related structures. *International Journal of Approximate Reasoning*.

[41] Abuhijleh E. A., Massa’deh M., Sheimat A., Alkouri A. (2021). Complex fuzzy groups based on rosenfeld’s approach. *WSEAS Transactions on Mathematics*.

[42] Bhunia S., Ghorai G., Ghorai G., Xin Q. (2021). On the characterization of Pythagorean fuzzy subgroups. *AIMS Mathematics*.

[43] Muhiuddin G., Mahboob A., Elnair M. E. (2022). A new study based on fuzzy bi-Γ-ideals in ordered-Γ-semigroups. *Journal of Computational and Cognitive Engineering*.

[44] John S. J., Suma P., Athira T. M. (2022). Multiset modules. *Journal of Computational and Cognitive Engineering*.

[45] Selvarathi M. (2021). Algebraic properties of implication-based intuitionistic fuzzy finite state machine over a finite group. *Journal of Discrete Mathematical Sciences and Cryptography*.

